# Supplementary material for: Fitting to the UK COVID-19 outbreak, short-term forecasts and estimating the reproductive number
Source: medRxiv. 2021 Jul 27:2020.08.04.20163782. Preprint. [Version 4] doi: 10.1101/2020.08.04.20163782 (PMC7430615; doi:10.1101/2020.08.04.20163782)
Supplement: 1 [file NIHPP2020.08.04.20163782V4-supplement-1.pdf]

medRxiv preprint doi: https://doi.org/10.1101/2020.08.04.20163782; this version posted July 27, 2021. The copyright holder for this preprint (which was not certified by peer review) is the author/funder, who has granted medRxiv a license to display the preprint in perpetuity.
It is made available under a CC-BY 4.0 International license .

SUPPLEMENTARY MATERIAL

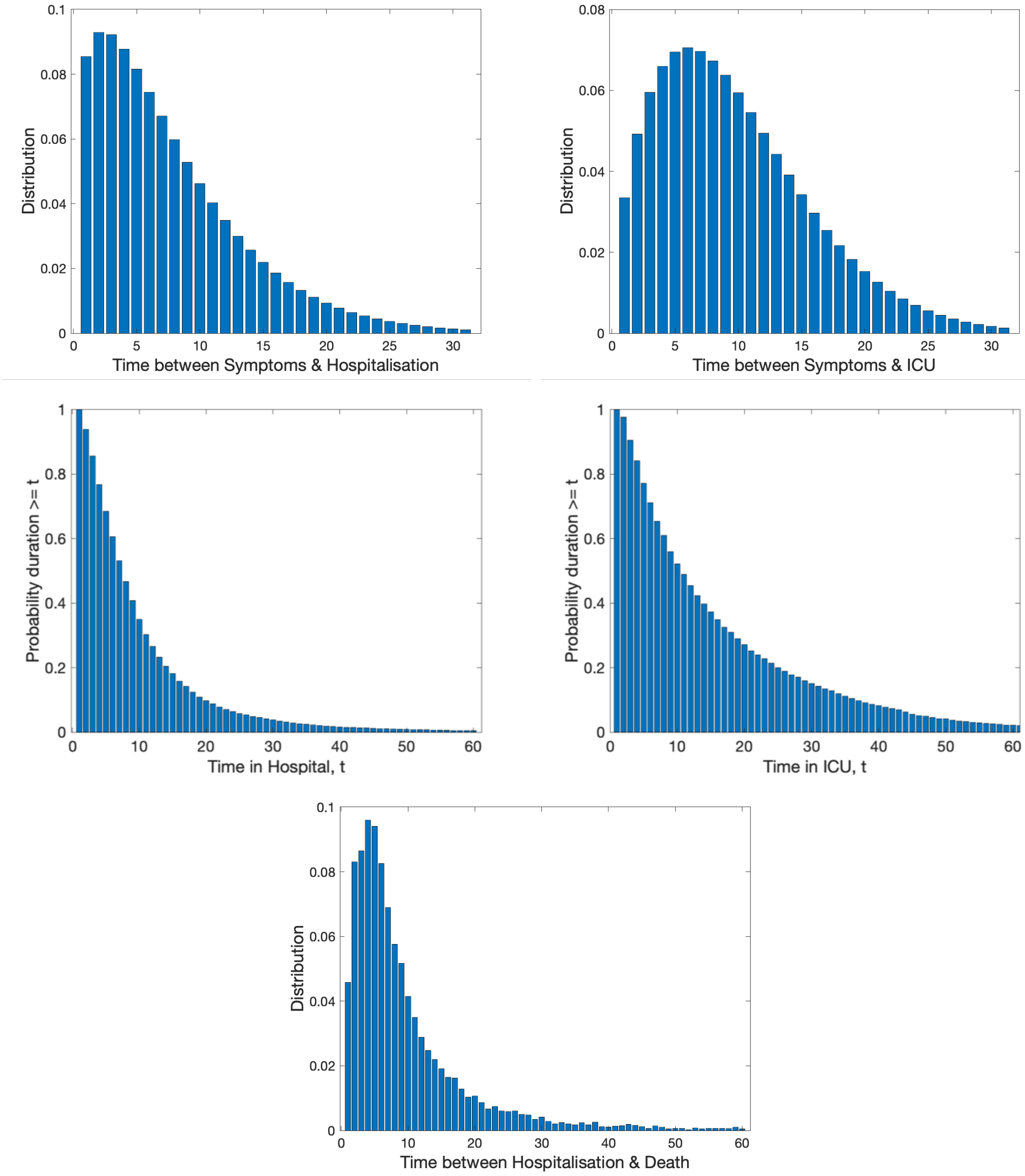

Fig. S1: Distribution of times and delays used in the model. (Top row, left) Time between symptom onset and hospitalisation, conditional on hospital admission occurring. (Top row, right) Time between symptom onset and ICU admittance, conditional on ICU admission occurring. We modelled these two delays as Weibull distributions estimated from the available data: Time between symptom onset and hospitalisation, $\lambda = 8.4$, $k = 1.4$; Time between symptom onset and ICU admittance, $\lambda = 10.9$, $k = 1.7$. We did not take these distributions directly from the available data which was relatively sparse due to the lack of certainty involving the date of symptom onset for many patients. (Middle row, left) Probability of the length of stay in hospital equalling or exceeding a duration of t days. (Middle row, right) Probability of the length of stay in ICU equalling or exceeding a duration of t days. (Bottom row) Time between hospitalisation and death. The remaining three distributions, displayed in the middle and bottoms rows, we took directly from the available data. All distributions are based on individual patient data as recorded by the COVID-19 Hospitalisation in England Surveillance System (CHESS) [21] and the ISARIC WHO Clinical Characterisation Protocol UK (CCP-UK) database sourced from the COVID-19 Clinical Information Network (CO-CIN) [22, 23].

medRxiv preprint doi: https://doi.org/10.1101/2020.08.04.20163782; this version posted July 27, 2021. The copyright holder for this preprint (which was not certified by peer review) is the author/funder, who has granted medRxiv a license to display the preprint in perpetuity.
It is made available under a CC-BY 4.0 International license .

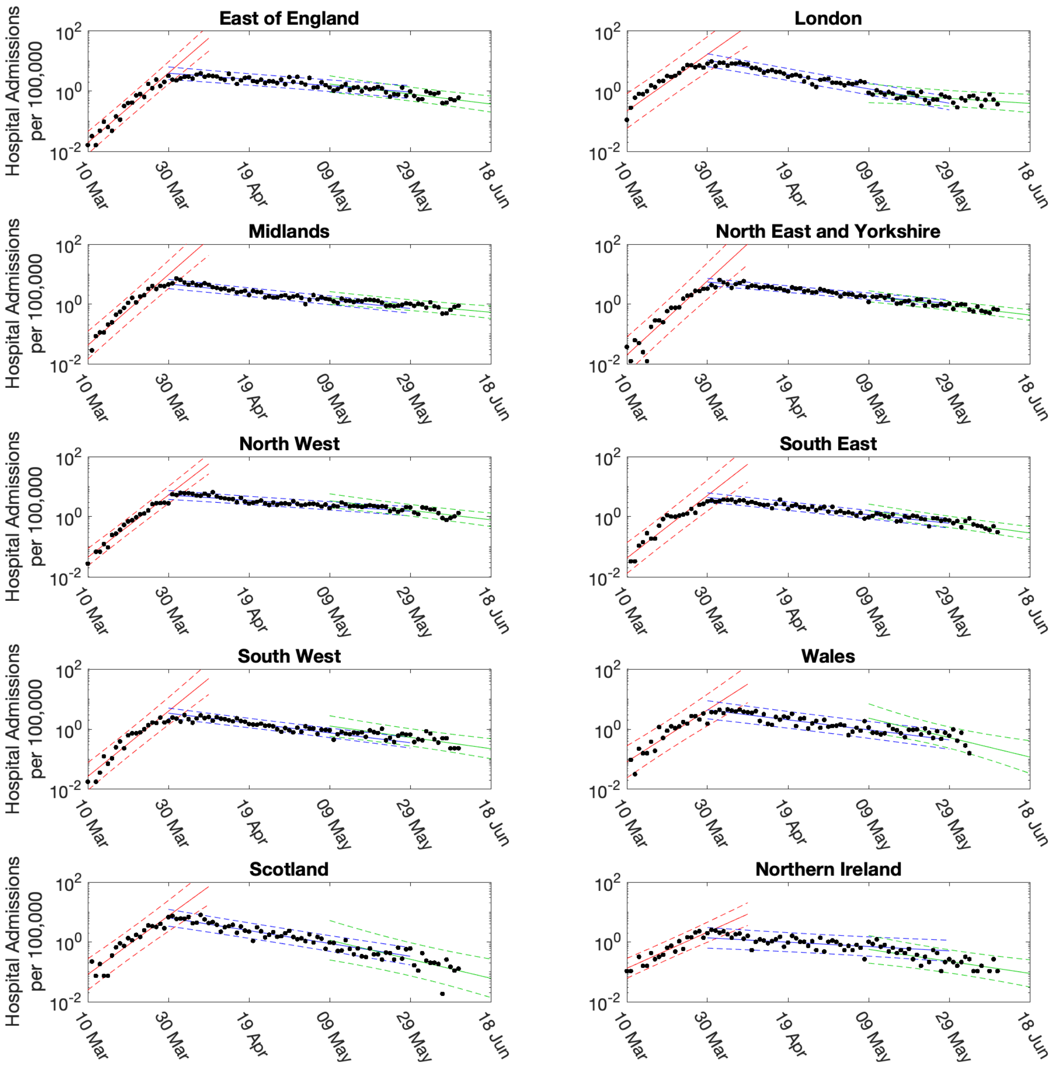

Fig. S2: Linear fits to daily hospital admissions per 100,000 individuals in each region. Points show the number of daily admissions to hospital (both in-patients testing positive and patients entering hospital following a positive test); results are plotted on a log scale. Three simple fits to the data are shown for pre-lockdown (red), strict-lockdown (blue) and relaxed-lockdown phases (green). Fits are a limit linear fit to the logged data (mean estimates depicted by solid lines, 95% confidence intervals by the dashed lines).

medRxiv preprint doi: https://doi.org/10.1101/2020.08.04.20163782; this version posted July 27, 2021. The copyright holder for this preprint
(which was not certified by peer review) is the author/funder, who has granted medRxiv a license to display the preprint in perpetuity.
It is made available under a CC-BY 4.0 International license .

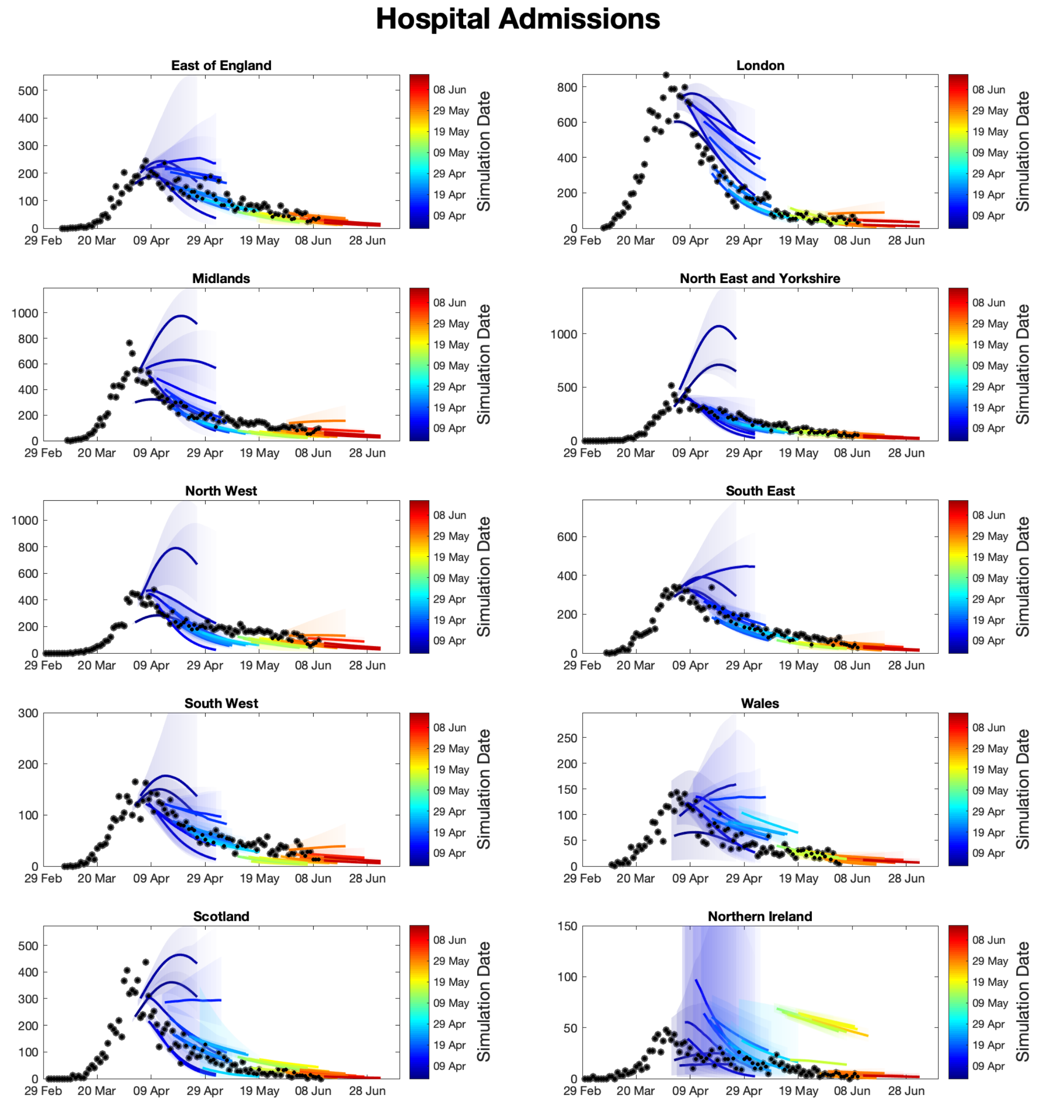

Fig. S3: Sequential comparison of daily hospital admission model results and data in each region. For all daily hospital admissions with COVID-19 in each region, we show the raw data (black dots) and a set of short-term predictions generated at different points during the outbreak. Changes to model fit reflect both improvements in model structure as well as increased amounts of data.

medRxiv preprint doi: https://doi.org/10.1101/2020.08.04.20163782; this version posted July 27, 2021. The copyright holder for this preprint (which was not certified by peer review) is the author/funder, who has granted medRxiv a license to display the preprint in perpetuity.
It is made available under a CC-BY 4.0 International license .

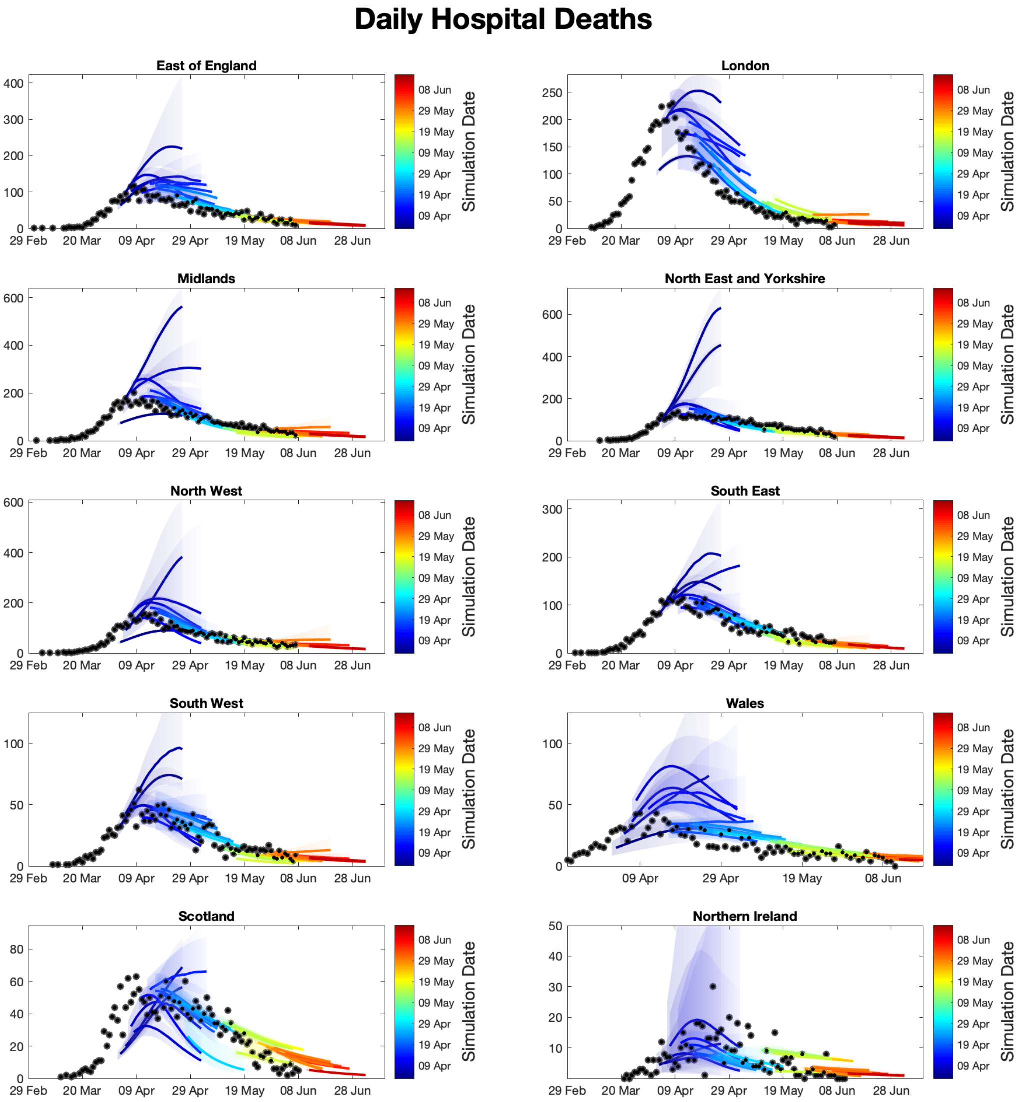

Fig. S4: Sequential comparison of daily hospital death model results and data in each region. For all daily hospital deaths with COVID-19 in each region, we show the raw data (black dots) and a set of short-term predictions generated at different points during the outbreak. Changes to model fit reflect both improvements in model structure as well as increased amounts of data.

medRxiv preprint doi: https://doi.org/10.1101/2020.08.04.20163782; this version posted July 27, 2021. The copyright holder for this preprint (which was not certified by peer review) is the author/funder, who has granted medRxiv a license to display the preprint in perpetuity.
It is made available under a CC-BY 4.0 International license .

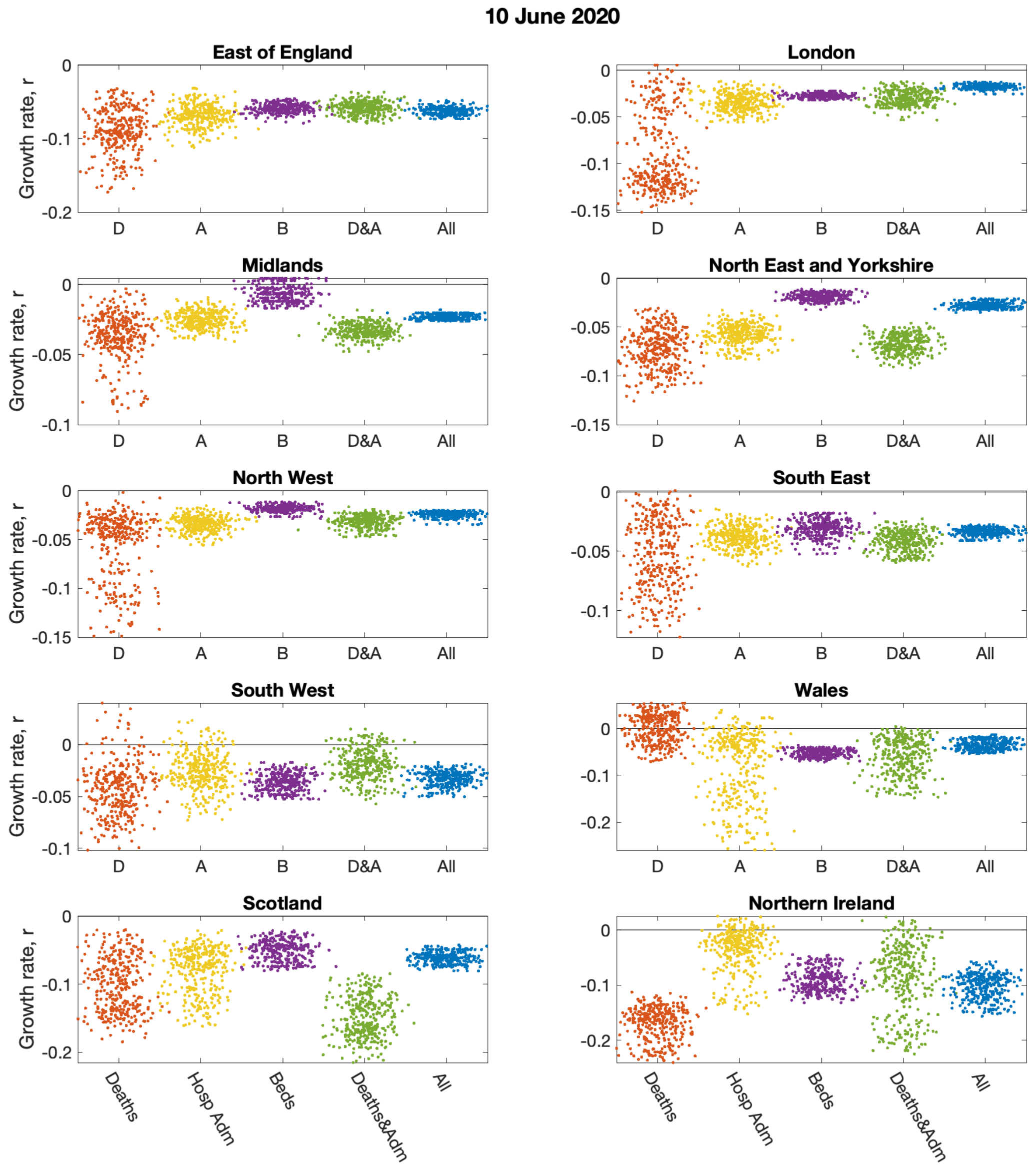

Fig. S5: Impact of data streams on estimated growth rates, by region. The impact on the regional growth rate (estimated from the ODE epidemic on 10th June 2020) of restricting the inference to different data streams (deaths only, hospital admissions, hospital bed occupancy, deaths and admissions or all data); the serology data was included in all inference. Parameters were inferred using data until 9th June 2020, while the r value comes from the change in predicted rate of change of new cases.

medRxiv preprint doi: https://doi.org/10.1101/2020.08.04.20163782; this version posted July 27, 2021. The copyright holder for this preprint (which was not certified by peer review) is the author/funder, who has granted medRxiv a license to display the preprint in perpetuity.
It is made available under a CC-BY 4.0 International license .

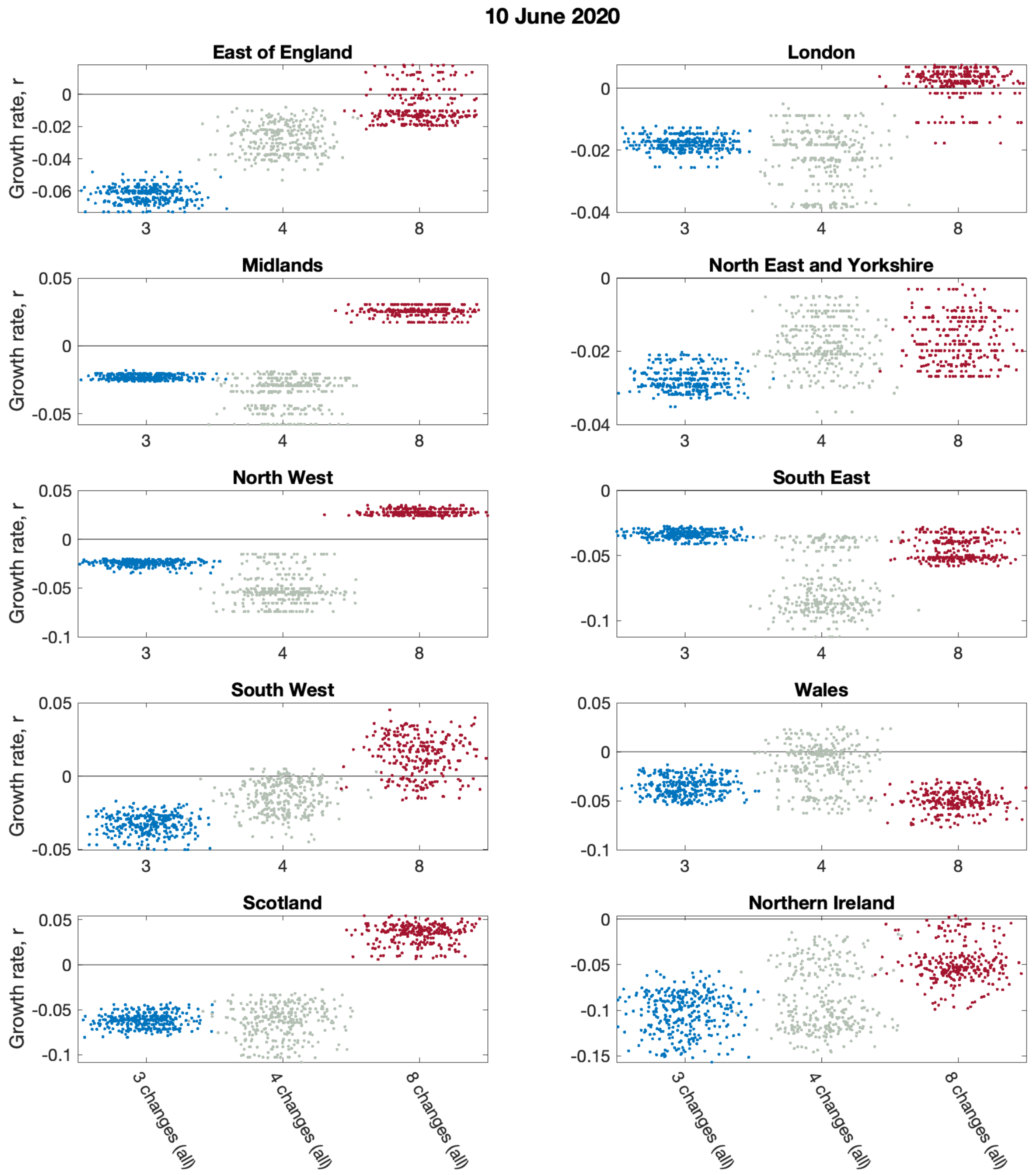

Fig. S6: Impact of model structure on estimated growth rates, by region. Analysis of having different numbers of lockdown phases on the estimated regional growth rate on 10th June 2020, while using all the data. The number of lockdown phases tested were there (blue dots), four (green dots) and eight (red dots). Parameters were inferred using data until 9th June 2020, while the r value comes from the change in predicted rate of change of new cases.

medRxiv preprint doi: https://doi.org/10.1101/2020.08.04.20163782; this version posted July 27, 2021. The copyright holder for this preprint (which was not certified by peer review) is the author/funder, who has granted medRxiv a license to display the preprint in perpetuity.
It is made available under a CC-BY 4.0 International license .

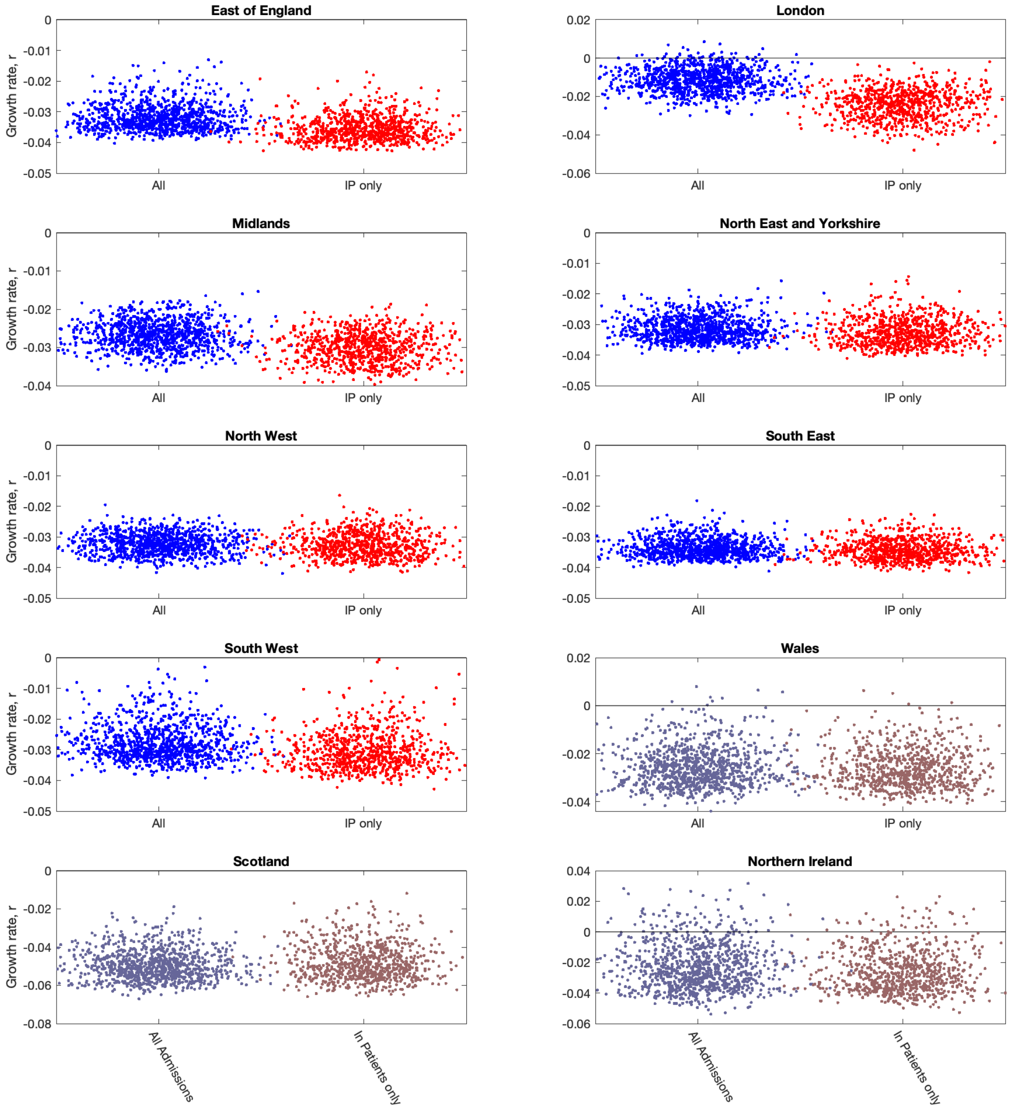

Fig. S7: Impact of including different types of hospital admission in parameter inference on the estimated regional growth rates (on 10th June 2020). For each region, growth rates were estimated from the ODE epidemic for 10th June 2020. In each panel, blue dots (on the left-hand side) give r estimates when using all hospital admissions in the parameter inference (together with deaths, ICU occupancy and serology when available); red dots (on the right-hand side) represent r estimates using an alternative inference method that restricted to fitting to in-patient hospital admission data (together with deaths, ICU occupancy and serology when available). Parameters were inferred using data until 9th June 2020, while the r value comes from the change in predicted rate of change of new cases. We observe that restricting the definition of hospital admission leads to a slight reduction in the growth rate r but a more pronounced reduction in the incidence. (This separation is not possible for the devolved nations, but the associated distributions are shown for completeness.)

medRxiv preprint doi: https://doi.org/10.1101/2020.08.04.20163782; this version posted July 27, 2021. The copyright holder for this preprint (which was not certified by peer review) is the author/funder, who has granted medRxiv a license to display the preprint in perpetuity.
It is made available under a CC-BY 4.0 International license .

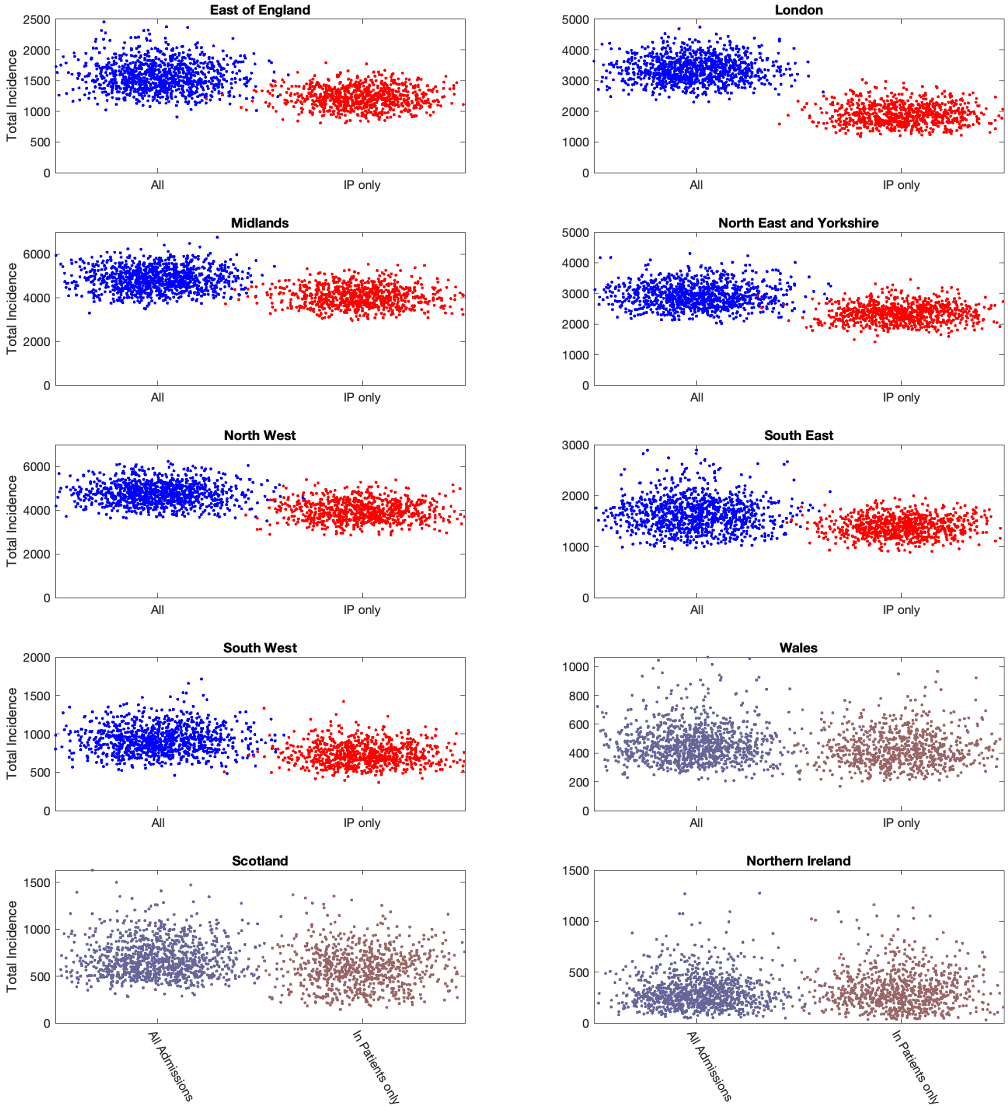

Fig. S8: Impact of including different types of hospital admission in parameter inference on the daily incidence (on 10th June 2020). For each region, daily incidences were estimated from the ODE epidemic for 10th June 2020. In each panel, blue dots (on the left-hand side) give incidence estimates when using all hospital admissions in the parameter inference (together with deaths, ICU occupancy and serology when available); red dots (on the right-hand side) represent incidence estimates using an alternative inference method that restricted to fitting to in-patient hospital admission data (together with deaths, ICU occupancy and serology when available). Parameters were inferred using data until 9th June 2020. We observe that restricting the definition of hospital admission leads to a pronounced reduction in the incidence. (This separation is not possible for the devolved nations, but the associated distributions are shown for completeness.)

medRxiv preprint doi: https://doi.org/10.1101/2020.08.04.20163782; this version posted July 27, 2021. The copyright holder for this preprint (which was not certified by peer review) is the author/funder, who has granted medRxiv a license to display the preprint in perpetuity.
It is made available under a CC-BY 4.0 International license .

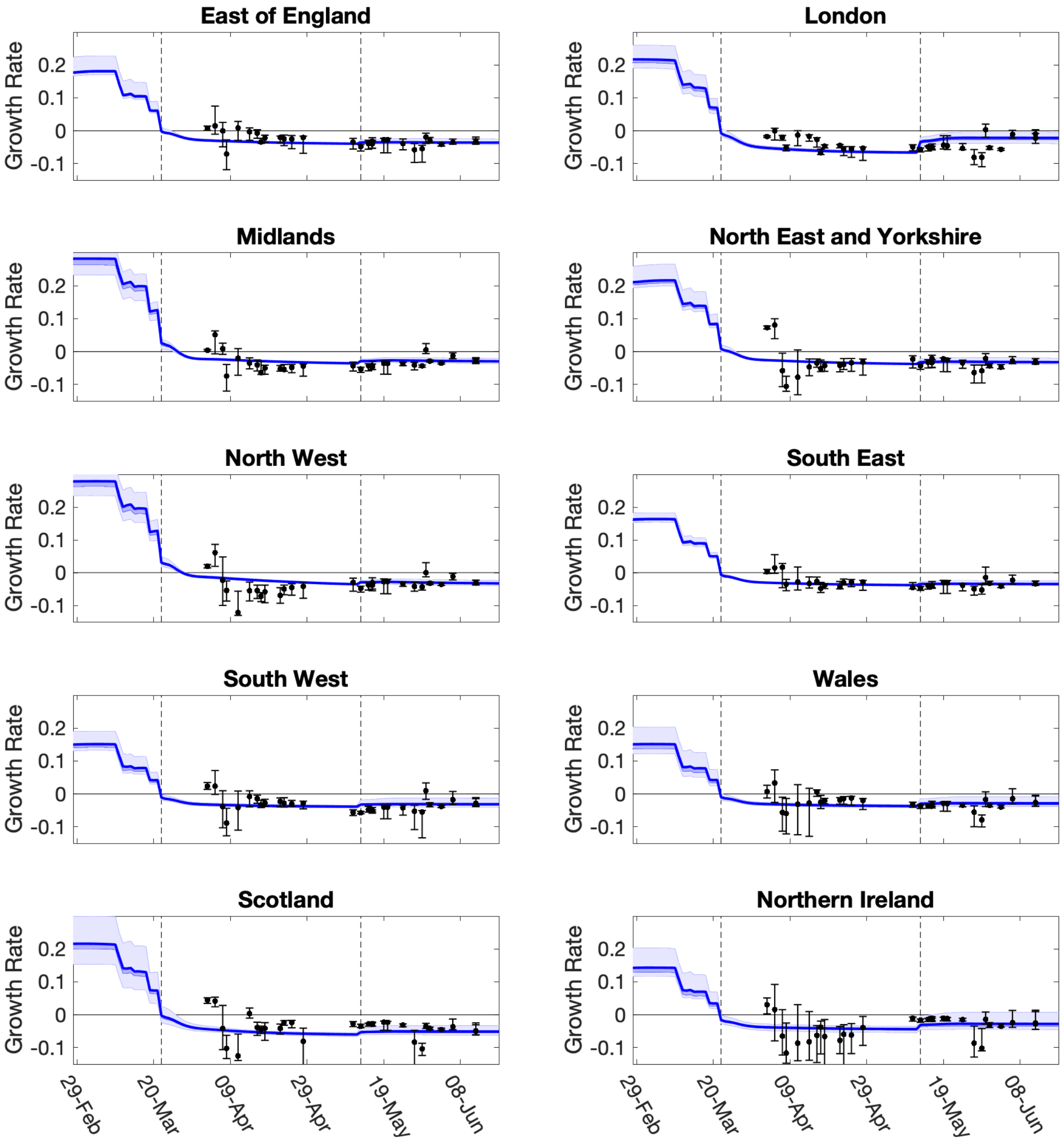

Fig. S9: Evolution of growth rate predictions and most recent model estimates in each of the ten regions. For each region, we show how predictions of r have evolved over time (dots and 95% credible intervals). These predictions are from the date the MCMC inference is performed. The solid blue line (together with 50% and 95% credible intervals) shows our estimate of r through time using the most recent fit to the data (performed on 14th June 2020 using in-patient data only). Vertical dashed lines show the two dates of main changes in policy, reflected in different regional ϕ values.

medRxiv preprint doi: https://doi.org/10.1101/2020.08.04.20163782; this version posted July 27, 2021. The copyright holder for this preprint (which was not certified by peer review) is the author/funder, who has granted medRxiv a license to display the preprint in perpetuity.
It is made available under a CC-BY 4.0 International license .

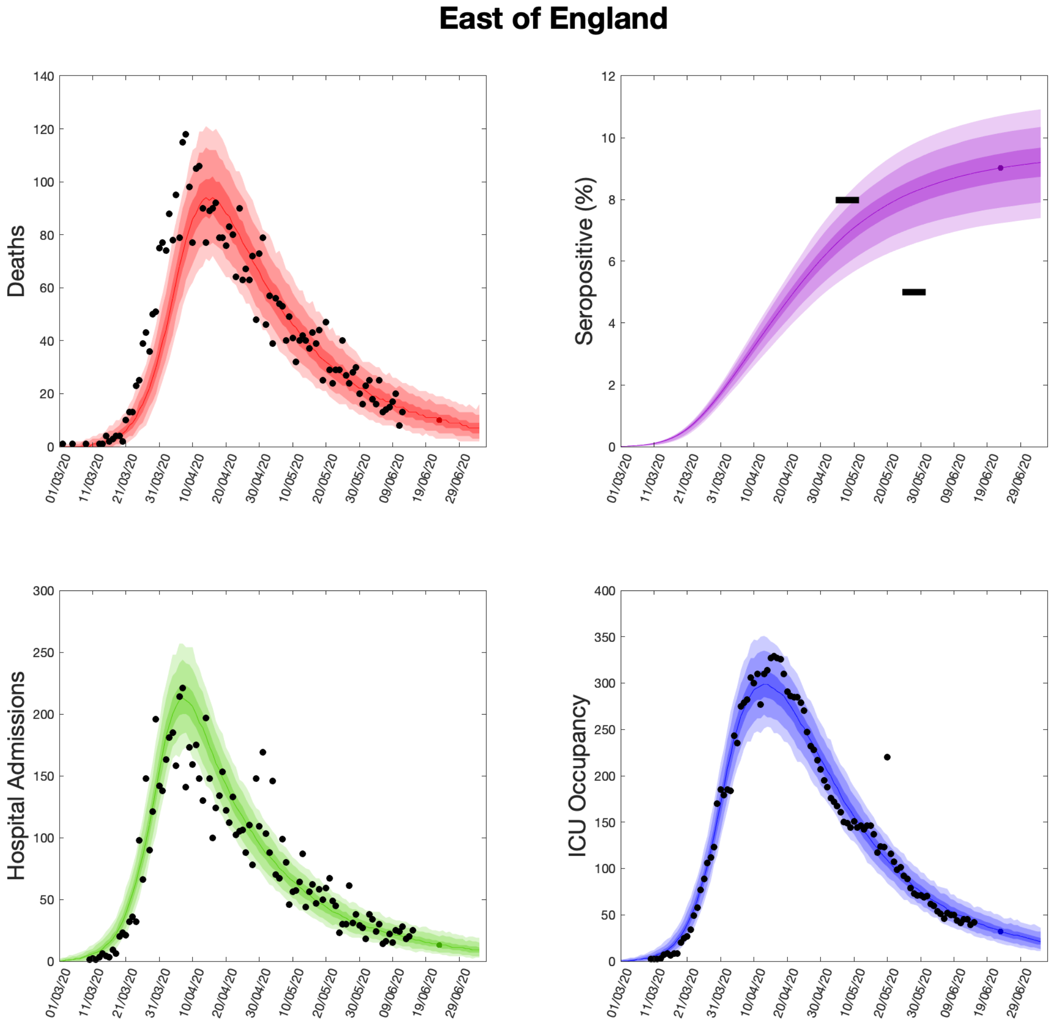

Fig. S10: Health outcome predictions of the ODE from the beginning of the outbreak and 3 weeks into the future for the East of England region. (Top left) Daily deaths; **(top right)** seropositivity percentage; **(bottom left)** daily hospital admissions; **(bottom right)** ICU occupancy. In each panel: filled markers correspond to observed data, solid lines correspond to the mean outbreak over a sample of posterior parameters; shaded regions depict prediction intervals, with darker shading representing a narrower range of uncertainty (dark shading - 50%, moderate shading - 90%, light shading - 99%). The intervals represent our confidence in the fitted ODE model, and do not account for either stochastic dynamics nor the observational distribution about the deterministic predictions - which would generate far wider intervals. Predictions were produced using data up to 14th June 2020.

medRxiv preprint doi: https://doi.org/10.1101/2020.08.04.20163782; this version posted July 27, 2021. The copyright holder for this preprint (which was not certified by peer review) is the author/funder, who has granted medRxiv a license to display the preprint in perpetuity.
It is made available under a CC-BY 4.0 International license .

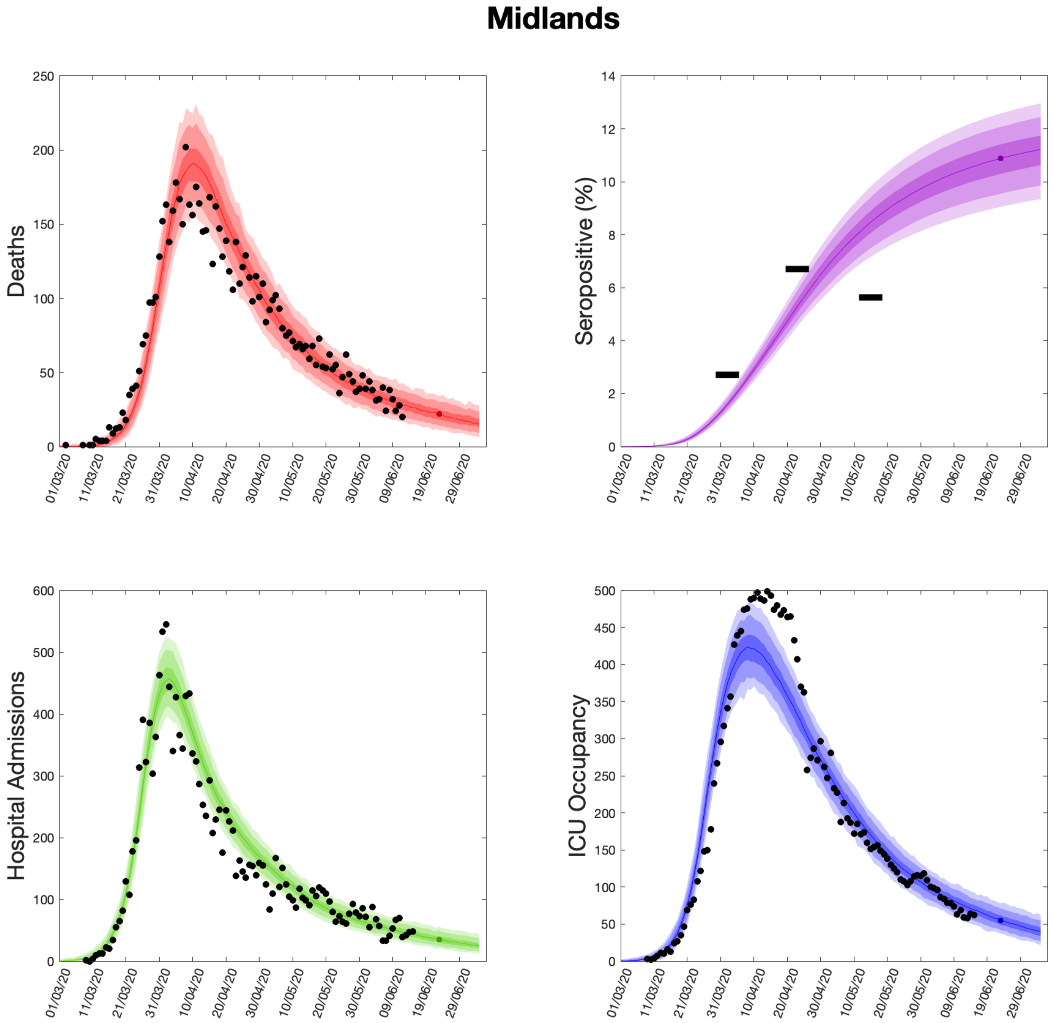

Fig. S11: Health outcome predictions of the ODE from the beginning of the outbreak and 3 weeks into the future for the Midlands region. (Top left) Daily deaths; **(top right)** seropositivity percentage; **(bottom left)** daily hospital admissions; **(bottom right)** ICU occupancy. In each panel: filled markers correspond to observed data, solid lines correspond to the mean outbreak over a sample of posterior parameters; shaded regions depict prediction intervals, with darker shading representing a narrower range of uncertainty (dark shading - 50%, moderate shading - 90%, light shading - 99%). The intervals represent our confidence in the fitted ODE model, and do not account for either stochastic dynamics nor the observational distribution about the deterministic predictions - which would generate far wider intervals. Predictions were produced using data up to 14th June 2020.

medRxiv preprint doi: https://doi.org/10.1101/2020.08.04.20163782; this version posted July 27, 2021. The copyright holder for this preprint (which was not certified by peer review) is the author/funder, who has granted medRxiv a license to display the preprint in perpetuity.
It is made available under a CC-BY 4.0 International license .

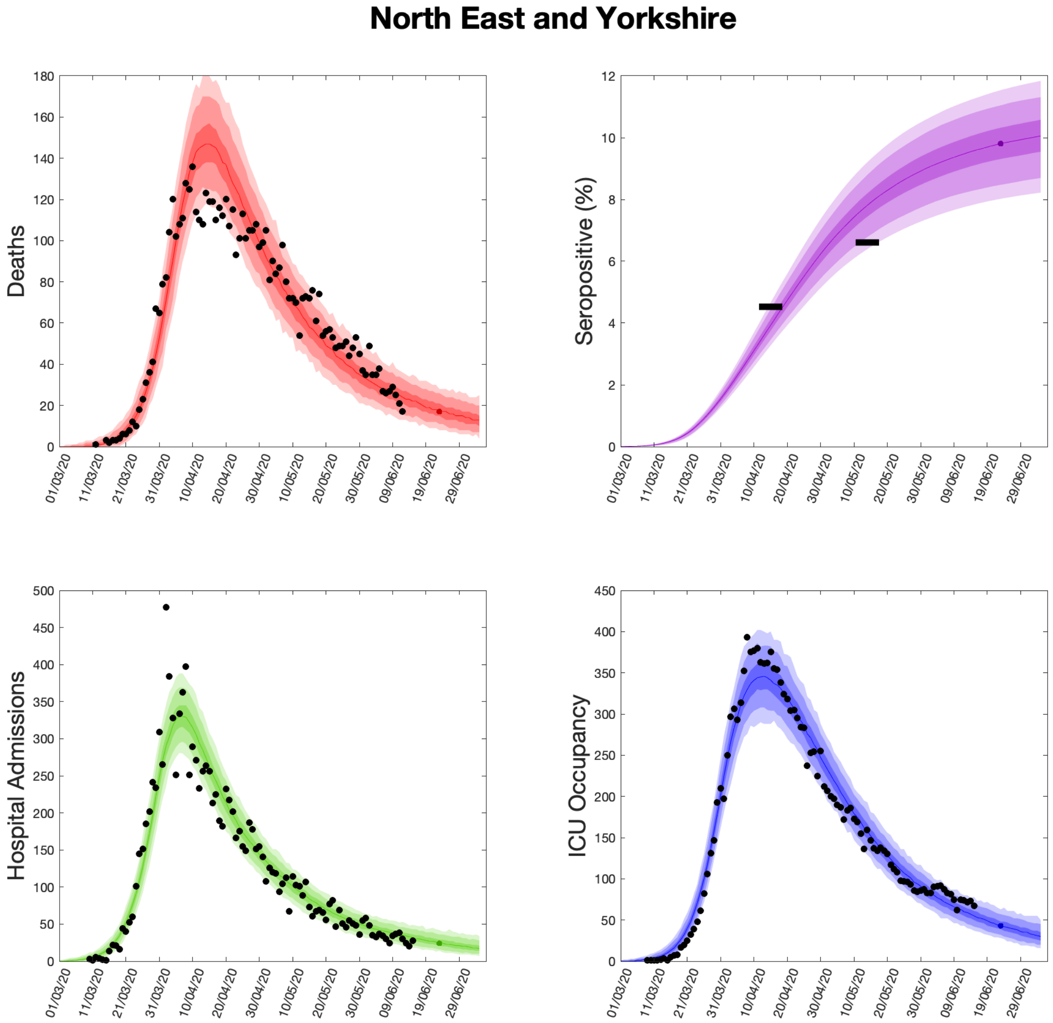

Fig. S12: Health outcome predictions of the ODE from the beginning of the outbreak and 3 weeks into the future for the North East & Yorkshire region. (Top left) Daily deaths; **(top right)** seropositivity percentage; **(bottom left)** daily hospital admissions; **(bottom right)** ICU occupancy. In each panel: filled markers correspond to observed data, solid lines correspond to the mean outbreak over a sample of posterior parameters; shaded regions depict prediction intervals, with darker shading representing a narrower range of uncertainty (dark shading - 50%, moderate shading - 90%, light shading - 99%). The intervals represent our confidence in the fitted ODE model, and do not account for either stochastic dynamics nor the observational distribution about the deterministic predictions - which would generate far wider intervals. Predictions were produced using data up to 14th June 2020.

medRxiv preprint doi: https://doi.org/10.1101/2020.08.04.20163782; this version posted July 27, 2021. The copyright holder for this preprint (which was not certified by peer review) is the author/funder, who has granted medRxiv a license to display the preprint in perpetuity.
It is made available under a CC-BY 4.0 International license .

North West

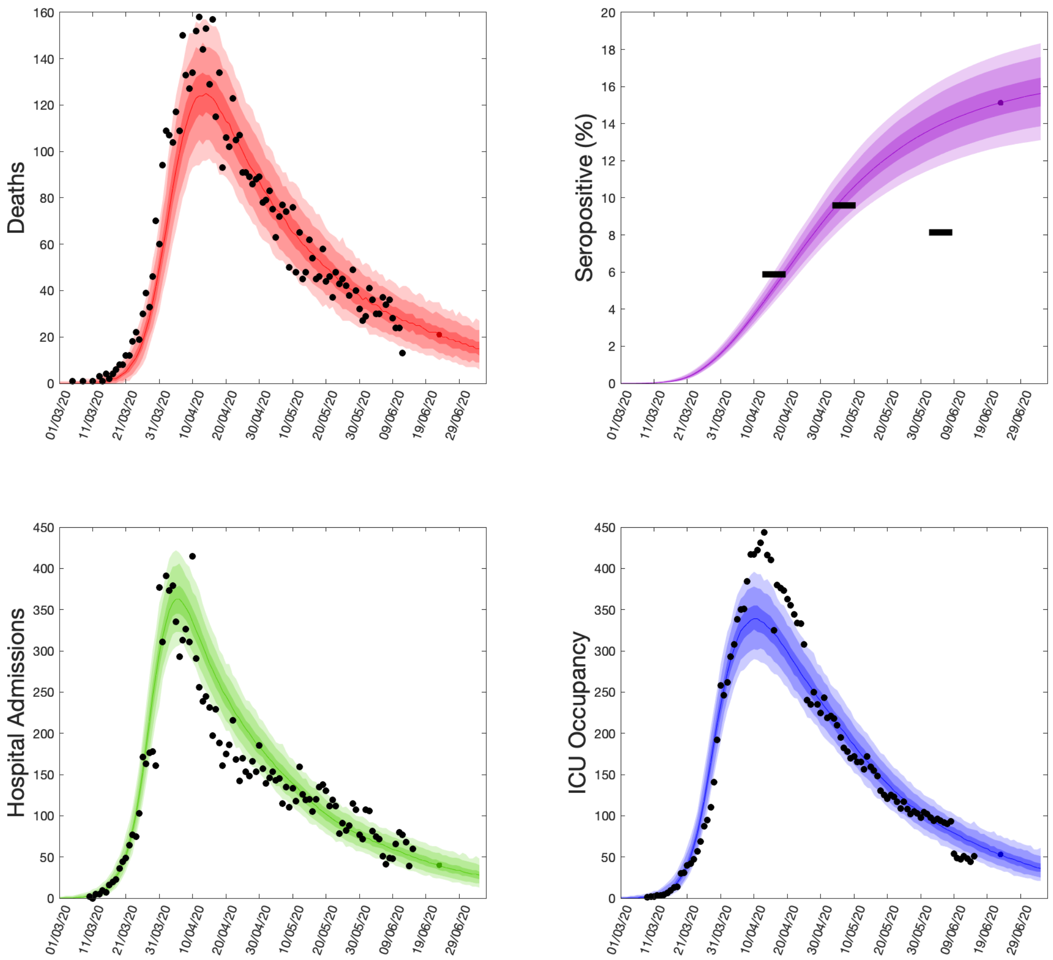

Fig. S13: Health outcome predictions of the ODE from the beginning of the outbreak and 3 weeks into the future for the North West region. (Top left) Daily deaths; **(top right)** seropositivity percentage; **(bottom left)** daily hospital admissions; **(bottom right)** ICU occupancy. In each panel: filled markers correspond to observed data, solid lines correspond to the mean outbreak over a sample of posterior parameters; shaded regions depict prediction intervals, with darker shading representing a narrower range of uncertainty (dark shading - 50%, moderate shading - 90%, light shading - 99%). The intervals represent our confidence in the fitted ODE model, and do not account for either stochastic dynamics nor the observational distribution about the deterministic predictions - which would generate far wider intervals. Predictions were produced using data up to 14th June 2020.

medRxiv preprint doi: https://doi.org/10.1101/2020.08.04.20163782; this version posted July 27, 2021. The copyright holder for this preprint (which was not certified by peer review) is the author/funder, who has granted medRxiv a license to display the preprint in perpetuity.
It is made available under a CC-BY 4.0 International license .

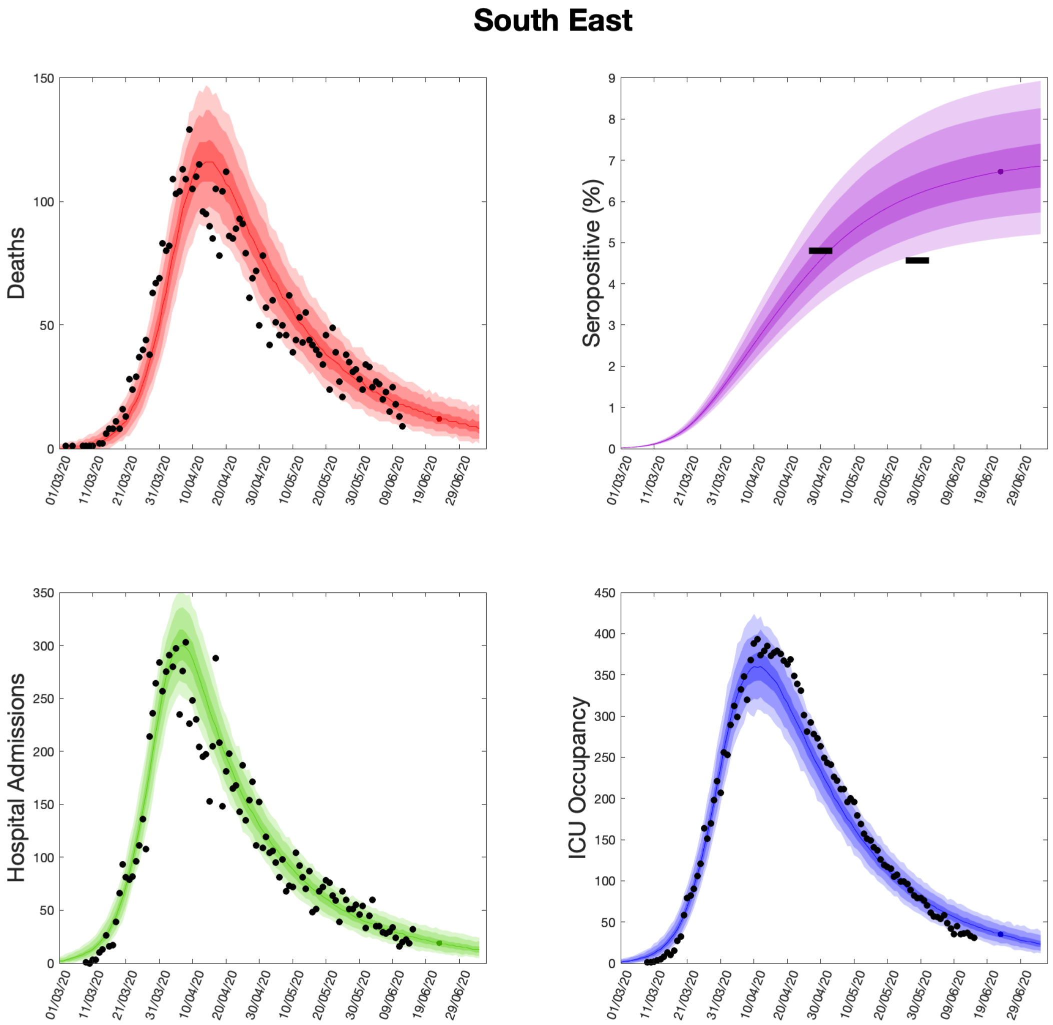

Fig. S14: Health outcome predictions of the ODE from the beginning of the outbreak and 3 weeks into the future for the South East region. (Top left) Daily deaths; **(top right)** seropositivity percentage; **(bottom left)** daily hospital admissions; **(bottom right)** ICU occupancy. In each panel: filled markers correspond to observed data, solid lines correspond to the mean outbreak over a sample of posterior parameters; shaded regions depict prediction intervals, with darker shading representing a narrower range of uncertainty (dark shading - 50%, moderate shading - 90%, light shading - 99%). The intervals represent our confidence in the fitted ODE model, and do not account for either stochastic dynamics nor the observational distribution about the deterministic predictions - which would generate far wider intervals. Predictions were produced using data up to 14th June 2020.

medRxiv preprint doi: https://doi.org/10.1101/2020.08.04.20163782; this version posted July 27, 2021. The copyright holder for this preprint (which was not certified by peer review) is the author/funder, who has granted medRxiv a license to display the preprint in perpetuity.
It is made available under a CC-BY 4.0 International license .

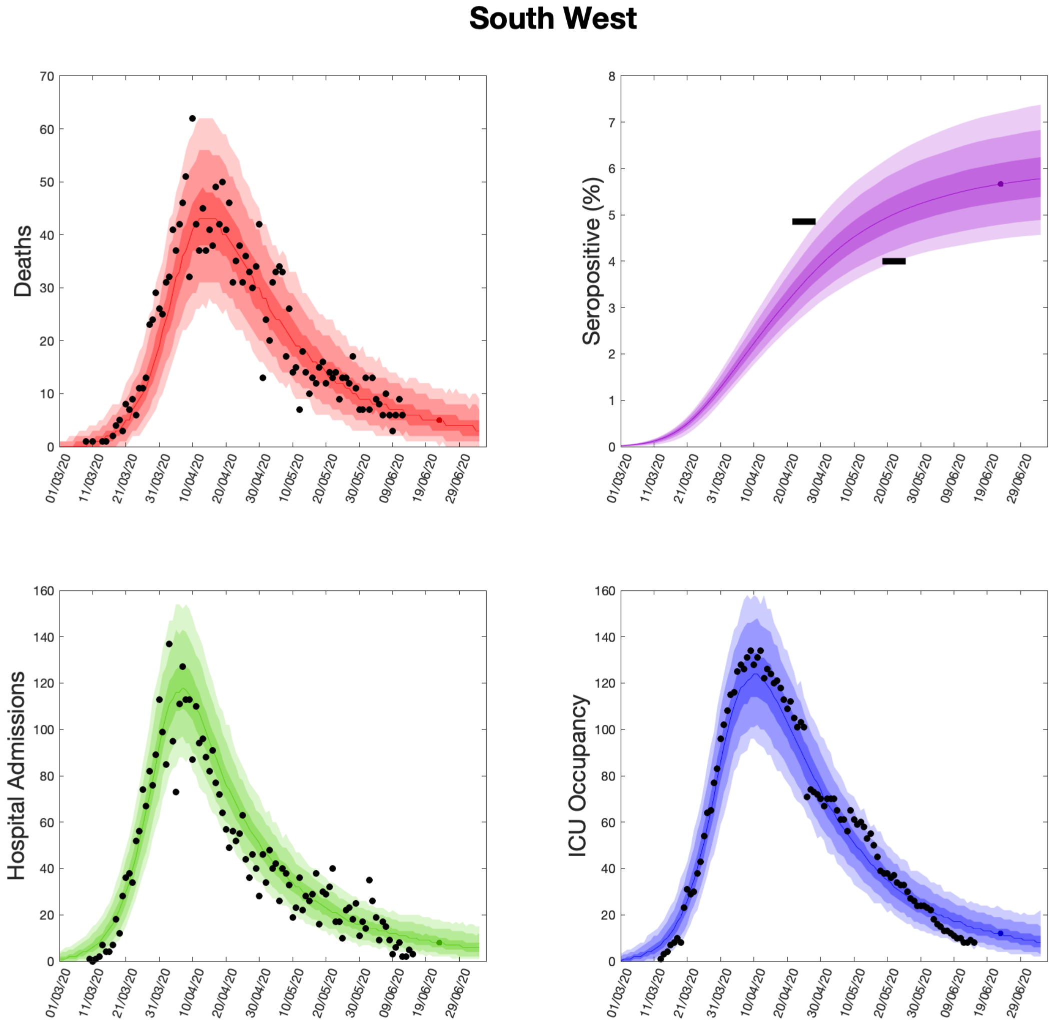

Fig. S15: Health outcome predictions of the ODE from the beginning of the outbreak and 3 weeks into the future for the South West region. (Top left) Daily deaths; **(top right)** seropositivity percentage; **(bottom left)** daily hospital admissions; **(bottom right)** ICU occupancy. In each panel: filled markers correspond to observed data, solid lines correspond to the mean outbreak over a sample of posterior parameters; shaded regions depict prediction intervals, with darker shading representing a narrower range of uncertainty (dark shading - 50%, moderate shading - 90%, light shading - 99%). The intervals represent our confidence in the fitted ODE model, and do not account for either stochastic dynamics nor the observational distribution about the deterministic predictions - which would generate far wider intervals. Predictions were produced using data up to 14th June 2020.

medRxiv preprint doi: https://doi.org/10.1101/2020.08.04.20163782; this version posted July 27, 2021. The copyright holder for this preprint (which was not certified by peer review) is the author/funder, who has granted medRxiv a license to display the preprint in perpetuity.
It is made available under a CC-BY 4.0 International license .

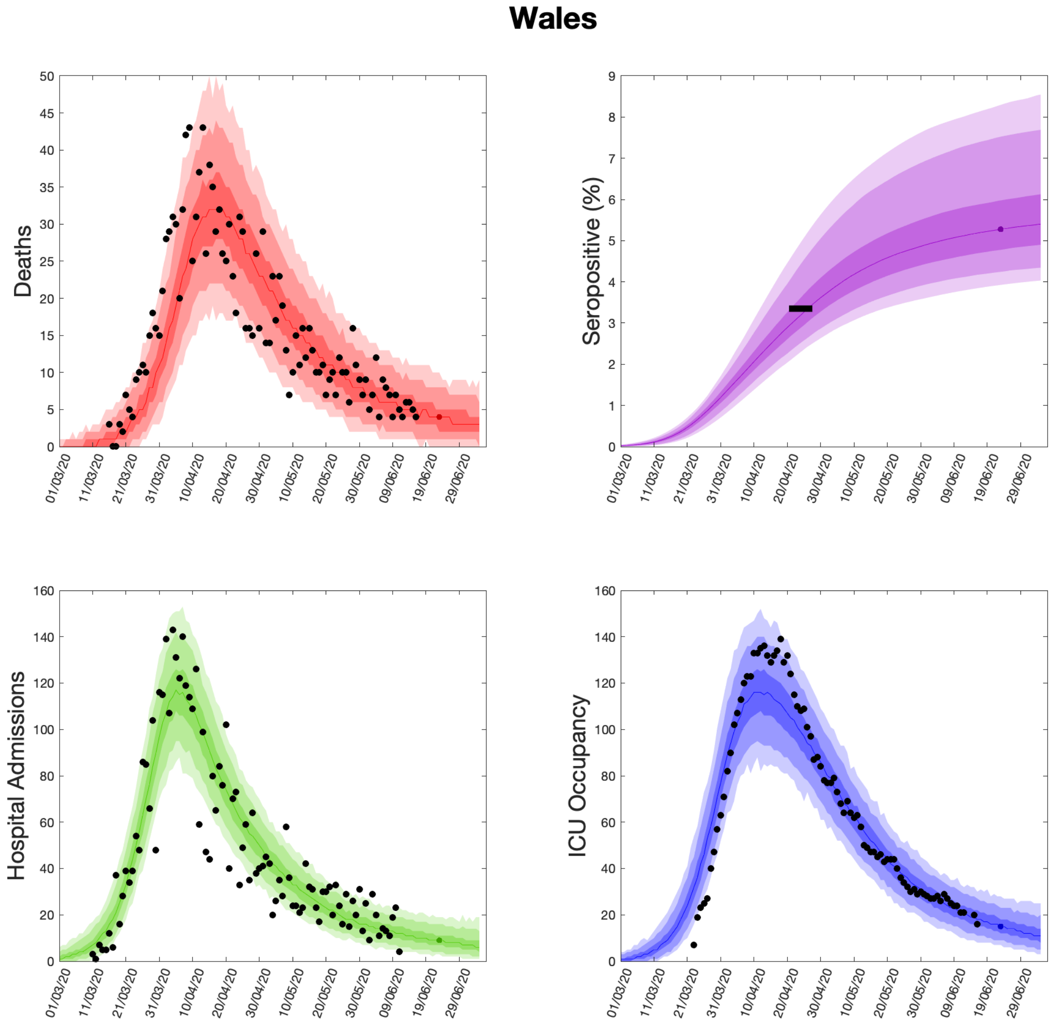

Fig. S16: Health outcome predictions of the ODE from the beginning of the outbreak and 3 weeks into the future for Wales. (Top left) Daily deaths; **(top right)** seropositivity percentage; **(bottom left)** daily hospital admissions; **(bottom right)** ICU occupancy. In each panel: filled markers correspond to observed data, solid lines correspond to the mean outbreak over a sample of posterior parameters; shaded regions depict prediction intervals, with darker shading representing a narrower range of uncertainty (dark shading - 50%, moderate shading - 90%, light shading - 99%). The intervals represent our confidence in the fitted ODE model, and do not account for either stochastic dynamics nor the observational distribution about the deterministic predictions - which would generate far wider intervals. Predictions were produced using data up to 14th June 2020.

medRxiv preprint doi: https://doi.org/10.1101/2020.08.04.20163782; this version posted July 27, 2021. The copyright holder for this preprint (which was not certified by peer review) is the author/funder, who has granted medRxiv a license to display the preprint in perpetuity.
It is made available under a CC-BY 4.0 International license .

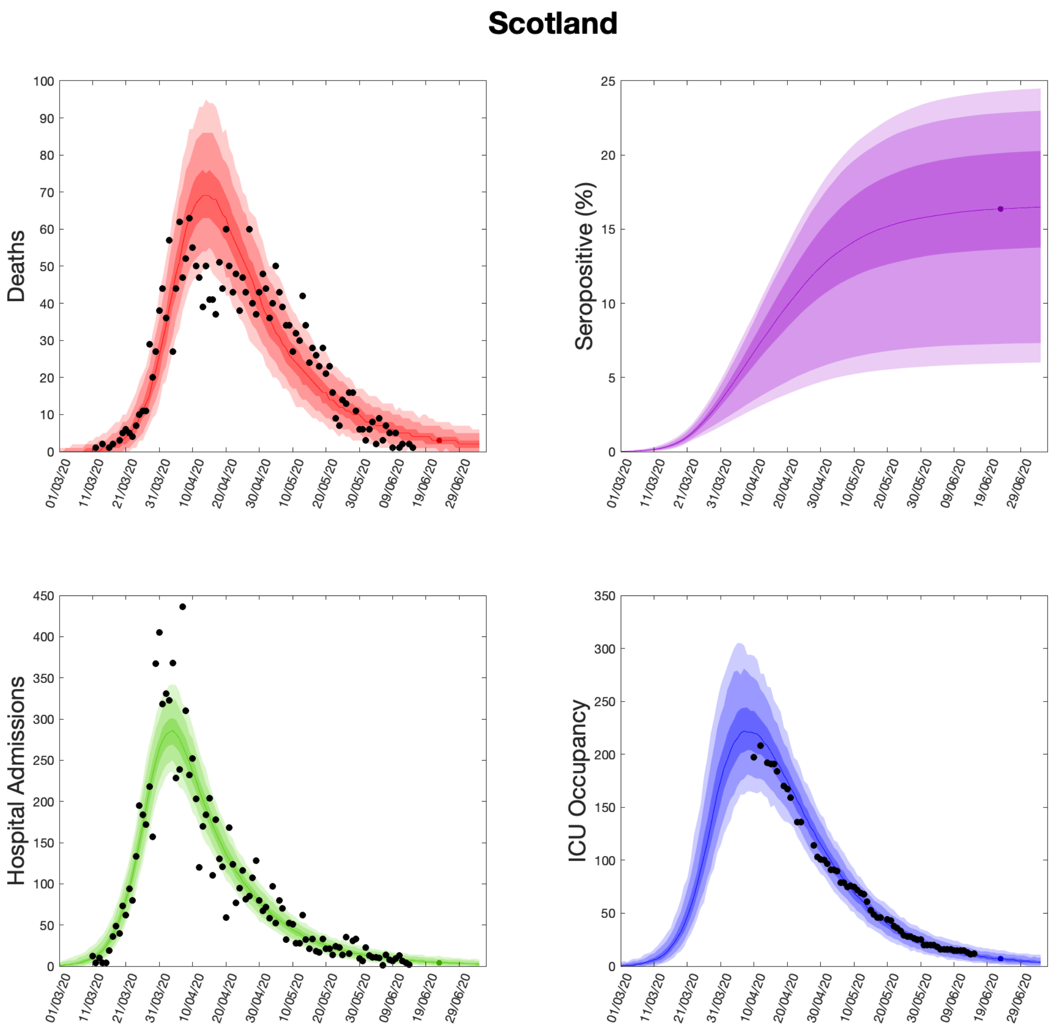

Fig. S17: Health outcome predictions of the ODE from the beginning of the outbreak and 3 weeks into the future for Scotland. (Top left) Daily deaths; **(top right)** seropositivity percentage; **(bottom left)** daily hospital admissions; **(bottom right)** ICU occupancy. In each panel: filled markers correspond to observed data, solid lines correspond to the mean outbreak over a sample of posterior parameters; shaded regions depict prediction intervals, with darker shading representing a narrower range of uncertainty (dark shading - 50%, moderate shading - 90%, light shading - 99%). The intervals represent our confidence in the fitted ODE model, and do not account for either stochastic dynamics nor the observational distribution about the deterministic predictions - which would generate far wider intervals. Predictions were produced using data up to 14th June 2020.

medRxiv preprint doi: https://doi.org/10.1101/2020.08.04.20163782; this version posted July 27, 2021. The copyright holder for this preprint (which was not certified by peer review) is the author/funder, who has granted medRxiv a license to display the preprint in perpetuity.
It is made available under a CC-BY 4.0 International license .

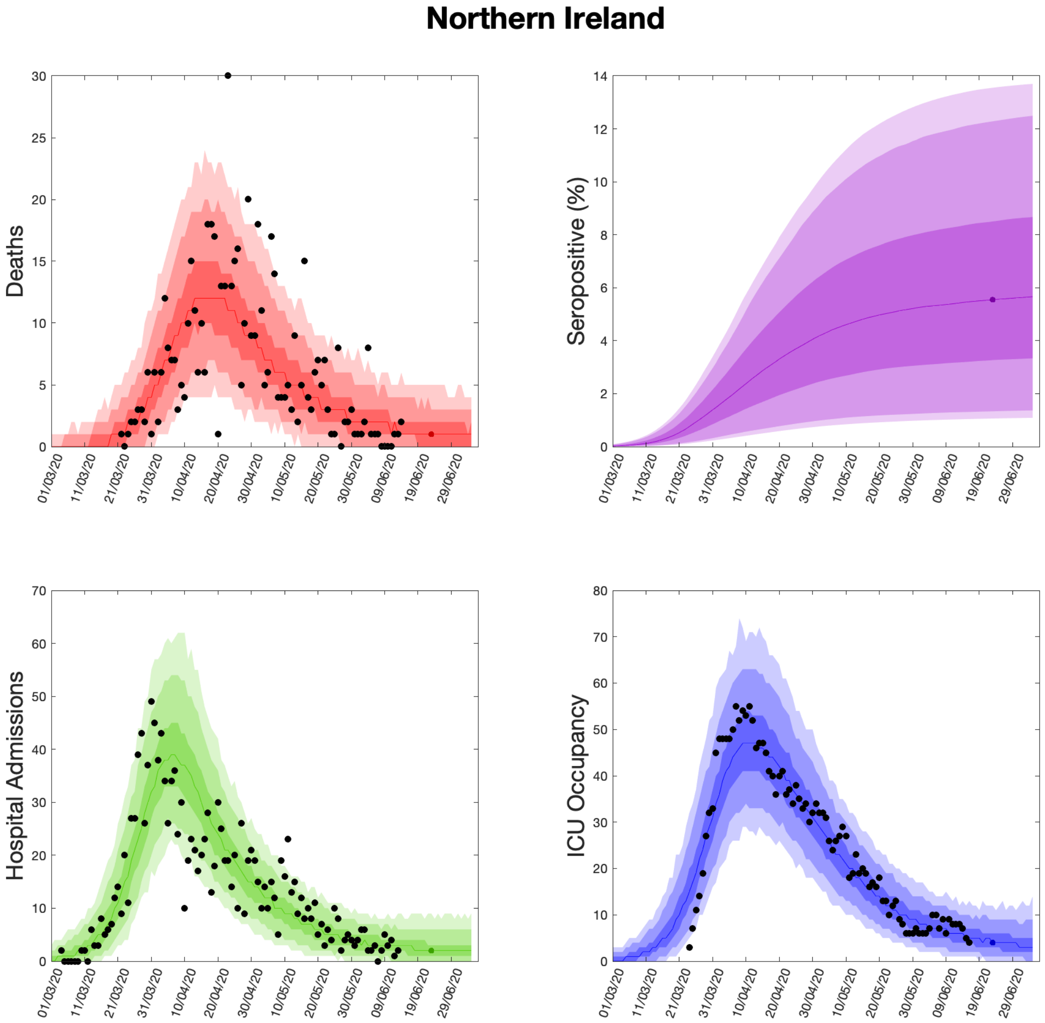

Fig. S18: Health outcome predictions of the ODE from the beginning of the outbreak and 3 weeks into the future for Northern Ireland. (Top left) Daily deaths; **(top right)** seropositivity percentage; **(bottom left)** daily hospital admissions; **(bottom right)** ICU occupancy. In each panel: filled markers correspond to observed data, solid lines correspond to the mean outbreak over a sample of posterior parameters; shaded regions depict prediction intervals, with darker shading representing a narrower range of uncertainty (dark shading - 50%, moderate shading - 90%, light shading - 99%). The intervals represent our confidence in the fitted ODE model, and do not account for either stochastic dynamics nor the observational distribution about the deterministic predictions - which would generate far wider intervals. Predictions were produced using data up to 14th June 2020.
